# Prospective analysis of video head impulse tests in patients with acute posterior circulation stroke

**DOI:** 10.3389/fneur.2023.1256826

**Published:** 2023-09-22

**Authors:** Sang Hee Ha, Dong Kyu Lee, Gayoung Park, Bum Joon Kim, Jun Young Chang, Dong-Wha Kang, Sun U. Kwon, Jong S. Kim, Hong Ju Park, Eun-Jae Lee

**Affiliations:** ^1^Department of Neurology, Asan Medical Center, University of Ulsan College of Medicine, Seoul, Republic of Korea; ^2^Department of Neurology, Gil Medical Center, Gachon University, Incheon, Republic of Korea; ^3^Department of Otolaryngology, Asan Medical Center, University of Ulsan College of Medicine, Seoul, Republic of Korea; ^4^Department of Neurology, Gangneung Asan Hospital, University of Ulsan, Gangneung, Gangwon-do, Republic of Korea

**Keywords:** corrective saccades, dorsal brainstem stroke, posterior circulation stroke, vestibular neuritis, vestibulo-ocular reflex, video head impulse test

## Abstract

**Background:**

Video head impulse tests (vHITs), assessing the vestibulo-ocular reflex (VOR), may be helpful in the differential diagnosis of acute dizziness. We aimed to investigate vHITs in patients with acute posterior circulation stroke (PCS) to examine whether these findings could exhibit significant abnormalities based on lesion locations, and to evaluate diagnostic value of vHIT in differentiating dizziness between PCS and vestibular neuritis (VN).

**Methods:**

We prospectively recruited consecutive 80 patients with acute PCS and analyzed vHIT findings according to the presence of dorsal brainstem stroke (DBS). We also compared vHIT findings between PCS patients with dizziness and a previously studied VN group (*n* = 29). Receiver operating characteristic (ROC) analysis was performed to assess the performance of VOR gain and its asymmetry in distinguishing dizziness between PCS and VN.

**Results:**

Patients with PCS underwent vHIT within a median of 2 days from stroke onset. Mean horizontal VOR gain was 0.97, and there was no significant difference between PCS patients with DBS (*n* = 15) and without (*n* = 65). None exhibited pathologic overt corrective saccades. When comparing the PCS group with dizziness (*n* = 40) to the VN group (*n* = 29), patients with VN demonstrated significantly lower mean VOR gains in the ipsilesional horizontal canals (1.00 vs. 0.57, *p* < 0.001). VOR gain and their asymmetry effectively differentiated dizziness in the PCS from VN groups, with an area under the ROC curve of 0.86 (95% CI 0.74–0.98) and 0.91 (95% CI 0.83–0.99, *p* < 0.001), respectively.

**Conclusion:**

Significantly abnormal vHIT results were rare in patients with acute PCS, even in the presence of DBS. Moreover, vHIT effectively differentiated dizziness between PCS and VN, highlighting its potential for aiding differential diagnosis of acute dizziness.

## Introduction

Fast and accurate diagnosis of stroke among patients presenting with acute dizziness is important. Stroke as a central cause of acute vestibular syndrome can be distinguished from peripheral causes using the bedside HINTS (head impulse test, nystagmus, test of skew) examination (1, 2). Especially, the head impulse test (HIT), which evaluates the vestibulo-ocular reflex (VOR), has been suggested as the best stroke predictor because an intact VOR during HIT may outperform even magnetic resonance imaging (MRI) in differentiating central from peripheral causes of acute vestibular syndrome (3, 4). However, bedside HIT may be technically demanding to non-experts, with interpretation varying across clinicians in the emergency department (5). Accordingly, the use of video head impulse test (vHIT), which can easily and objectively measure VOR, has been suggested as an “ECG for the eyes” and has showed promising results (1).

Decreases in VOR gain and subsequent catch-up saccades develop after damages to the peripheral vestibular system with disruption of vestibular input signals (6, 7). The VOR is mediated by various parts of the dorsal brainstem, which includes central structures, such as the medial longitudinal fasciculus (MLF) and the nuclei of the cranial nerves III, IV, and VI (8). Central lesions affecting these structures can also induce VOR impairment (9). Recent studies that evaluated HITs with magnetic search coils or video-based techniques have reported frequent VOR abnormalities in patients with central vestibular disorders (8, 10). These findings raise questions on whether vHIT can be used as a screening tool to differentiate between central and peripheral causes of acute dizziness in clinical settings.

In this prospective study, we aimed to investigate the vHITs in consecutive patients with acute posterior circulation stroke (PCS). First, we determined whether these findings could show significant abnormalities according to the presence of dorsal brainstem stroke (DBS). Additionally, we evaluated whether VOR gains in patients with DBS differ along the longitudinal axis of brainstem (i.e., midbrain vs. pons vs. medulla), because the vestibular pathway spreads out and becomes sparse as it ascends from the caudal to the rostral part. Finally, we evaluated the diagnostic value of vHITs in the differential diagnosis of dizziness between PCS and acute vestibular neuritis (VN).

## Materials and methods

### Patient selection

Of the consecutive patients who were admitted to the stroke unit of Asan Medical Center (Seoul, South Korea) between July 2021 and April 2022, we prospectively recruited those affected by acute PCS (within 24 h of stroke onset) with mild neurologic deficits [National Institutes of Health Stroke Scale (NIHSS) score ≤ 7] (11). Because we aimed to comprehensively analyze vHIT results in acute stroke patients with lesions in the posterior circulation territory, we included patients regardless of the presence of acute dizziness. We excluded individuals with concomitant acute lesions in the anterior circulation, as well as those exhibiting altered mental status (characterized by disorientation, confusion, or diagnosed loss of consciousness by neurologists), and those with active systemic diseases (such as infection or active cancer) (12). These exclusions were implemented due to the potential for compromised cooperation and safety concerns during the testing procedure. The included patients underwent vHIT after the stabilization of neurologic symptoms, which were defined at the discretion of attending physicians.

We also reviewed patients with VN diagnosed in our institution between March 2014 and December 2014 as the control group (13). These patients were enrolled in a randomized controlled 98 trial and prospectively underwent vHIT (13). Patients with VN were included as following criteria: (1) presenting with acute persistent vertigo with unidirectional nystagmus without hearing loss of middle ear pathology on clinical examination, (2) had undergone both vHIT and caloric test finding (abnormal finding: canal paresis ≥ 20%), (3) and no central vestibular diseases from neurological examination or magnetic resonance imaging (13). The study protocol was approved by the local ethics committee (IRB approval number: 2021-0901), and all patients provided written informed consent.

### Clinical and imaging assessments

Demographic information and risk factors, along with the chief symptom of acute dizziness were obtained at admission. The symptoms of acute dizziness were defined as follows: (1) vertigo, which is an illusion of movement, either of the person or the visual surrounding, (2) dysequilibrium without vertigo, (3) presyncope (near-faint), and (4) psychophysiological dizziness, which is often associated with anxiety and panic (14). Examinations were conducted by an experienced neurologist. The cause of stroke was categorized according to the Trial of Org 10,172 in Acute Stroke Treatment (TOAST) classification (15). Neurological deficit caused by stroke was evaluated based on the NIHSS score at admission.

All patients underwent MRI using a 3.0-T Philips scanner (Philips Healthcare, Eindhoven, Netherlands) that included diffusion-weighted imaging and MR angiography. The locations of acute lesions were evaluated using diffusion-weighted imaging and classified as follows: cerebellar [superior cerebellar, anterior inferior cerebellar, and posterior inferior cerebellar (PICA) artery territory], medulla oblongata, pons, midbrain, thalamus, and medial tempo-occipital areas. If a patient had ischemic lesions in more than one location, each location was classified as a separate location variable; thus, a single patient could have multiple values for the location of stroke lesions. DBS was defined as the presence of lesions in the dorsal medulla oblongata or tegmentum of the midbrain and pons, which are related to the central vestibular pathway (16). If a patient had DBS, they were categorized into the DBS group irrespective of the presence of lesions outside of the dorsal brainstem area. Lesion locations were consensually determined by two stroke experts (SH Ha and E.-J Lee) who were blinded to clinical information.

### Video head impulse test

Recording of the head and eye movements were conducted using vHIT (SLVNG, SLMED, Seoul, South Korea) for PCS patients and ICS Impulse 3-D vHIT system (GN Otometrics, Taastrup, Denmark) for VN patients. Despite the variation in the machines and examiners, the procedures adhered to the protocol established by our institution. The subjects were seated in a height-adjustable chair which allowed the examiner to adjust the height of each subject’s head to an optimal level for the examination. After calibration, an experienced well-trained technician delivered repetitive passive and rapid head rotations (head rotation: 10°–15°, peak velocity:150°/s–250°/s, duration 150–200 ms) in horizontal and vertical canals with minimum of 10 times in each plane. The device records head and eye velocity traces. VOR gain values were derived by calculating the ratio of the area under the eye velocity curve with respect to the area under the head velocity curve. To reduce artifacts, peak velocities were maintained above 150°/s, and trace oscillations during head movement, pseudo-saccades (blinks), head-motion artifacts, and other tracking errors were excluded from the analysis. Also, neurological saccadic eye movements may interfere with the interpretation of vHIT data. Therefore, if a patient was not cooperative enough to reliably undergo the test, the result was recorded as not applicable (17).

Abnormal VOR gain was defined as a lateral gain <0.8 for lateral canals and a posterior/anterior gain < 0.7 for anterior and posterior canals (5, 18). Gain asymmetry (GA) was evaluated in the plane of the lateral semicircular canal and calculated using a general formula analogous to the formula for caloric canal paresis:


GA=[(Gc−Gi)/(Gc+Gi)]×100%

where *G*c is the vHIT gain for head impulses exciting the contralateral canal and *G*i is the vHIT gain for head impulses exciting the ipsilateral canal (18). Corrective saccades were classified as covert or overt if they occurred before or after the end of the head movement, respectively (19). When comparing between VN and PCS patients, VOR gain was calculated based on the side of the lesion, specifically ipsilesional and contralesional. The definition of ipsilesional VOR gain in stroke patients was derived by considering the direction of the diminished VOR gain value.

### Statistical analysis

Clinical characteristics and vHIT findings were compared in patients with PCS according to the presence of DBS. Also, VOR gains were compared in patients with DBS according to the caudal-to-rostral distribution (i.e., medulla, pons, and midbrain). Furthermore, we analyzed vHIT findings between PCS with acute dizziness and VN groups. Fisher’s exact test was used to analyze categorical variables, and Student’s *t*-test or the Mann–Whitney *U*-test was used to analyze continuous variables, as appropriate.

To evaluate the diagnostic performance of vHIT, we conducted receiver operating characteristic (ROC) analysis for VOR gain and VOR gain asymmetry to ascertain sensitivity and specificity, as well as to identify the optimal threshold values. The IBM SPSS Statistics software, version 21.0 (IBM Corp., Armonk, NY, United States) was used for all analyses. A value of *p* < 0.05 was considered statistically significant.

## Results

### Baseline characteristics

**Figure 1:**
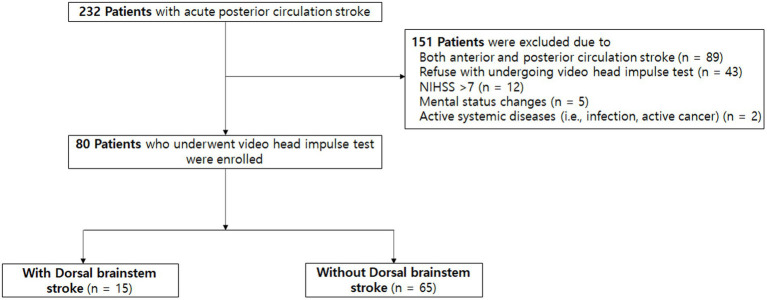
Study flow chart. NIHSS indicates National Institutes of Health Stroke Scale.

During the study period, a total of 232 patients with acute PCS were admitted to our center, and 80 (34.5%) patients satisfied the inclusion criteria (Figure 1). The mean age was 65 ± 13 years, 57 (71.3%) patients were men, and the median initial NIHSS score was 2 [interquartile range (IQR), 0–3; Table 1]. Among them, 40 (50.0%) patients had the symptom of acute dizziness. The most common lesion location was posterior inferior cerebellar arterial territory (35.8%), while multiple lesions were observed in 24 (30.0%) patients. All patients were confirmed as stroke with MRI conducted within 24 h following the symptom onset.

**Table 1:** Baseline characteristics of PCS patients.

	Total *n* = 80	With dorsal brainstem stroke *n* = 15	Without dorsal brainstem stroke *n* = 65	Value of *p*
Age (years)	65 ± 13	62 ± 14	66 ± 13	0.321
Male sex	57 (71.3)	9 (60.0)	48 (73.8)	0.286
Hypertension	56 (70.0)	12 (80.0)	44 (67.7)	0.348
Diabetes mellitus	33 (41.3)	7 (46.7)	26 (40.0)	0.636
Hyperlipidemia	41 (51.2)	12 (80.0)	29 (44.6)	0.013
Atrial fibrillation	16 (20.0)	3 (20.0)	13 (20.0)	>0.99
Smoking	33 (41.3)	4 (26.7)	29 (44.6)	0.203
History of previous stroke	23 (28.7)	3 (20.0)	20 (30.8)	0.406
History of peripheral vertigo	0 (0)	0 (0)	0 (0)	NA
Initial NIHSS score	2 (0–3)	3 (1–3)	2 (0–4)	0.809
TOAST classification				0.647
Larger artery disease	17 (21.3)	3 (20.0)	14 (21.5)	
Small vessel disease	20 (25.0)	5 (33.3)	15 (23.1)	
Cardioembolism	12 (15.0)	1 (6.7)	11 (16.9)	
Other determined	21 (26.3)	3 (20.0)	18 (27.7)	
Undetermined	10 (12.5)	3 (20.0)	7 (10.8)	
**Symptom**				
Acute dizziness	40 (50.0)	12 (80.0)	28 (43.1)	0.010
**Lesion location**				
Temporo-occipital	20 (24.7)	0 (0)	20 (30.8)	0.013
Thalamus	20 (24.7)	0 (0)	20 (30.8)	0.013
Midbrain	6 (7.4)	5 (33.3)	1 (1.5)	<0.001
Pons	18 (22.2)	6 (40.0)	12 (18.5)	0.072
Medulla oblongata	4 (4.9)	4 (26.7)	0 (0)	<0.001
Cerebellar				
SCA	10 (12.3)	0 (0)	10 (15.4)	0.104
AICA	1 (1.2)	0 (0)	1 (1.5)	0.629
PICA	29 (35.8)	3 (20.0)	27 (41.5)	0.120
**Multiple lesions**	24 (30.0)	3 (20.0)	21 (32.3)	0.348

Results are presented as numbers and percentages or as means ± standard deviations or median (interquartile ranges). NIHSS, National Institutes of Health Stroke Scale; TOAST, Trial of Org 10,172 in Acute Stroke Treatment; AICA, anterior inferior cerebellar artery; PICA, posterior inferior cerebellar artery; SCA, superior cerebellar artery.

DBS was observed in 15 (18.8%) patients. The most common lesion location was the midbrain (*n* = 5; 33.3%), followed by the medulla oblongata (*n* = 4; 26.7%). Demographic and risk factors were not significantly different between patients with and without DBS except for hyperlipidemia (12 [80.0%] vs. 29 [44.6%]; *p* = 0.013) and the symptoms of acute dizziness (12 [80.0%] vs. 28 [43.1%]; p = 0.01), which were more common in patients with DBS.

### Examination and vHIT findings

Skew deviation was detected in 6 patients (7.5%), while extraocular movement limitation was observed in 3 patients (3.8%). Both findings were notably more prevalent in patients with dorsal brainstem (DBS) involvement (Table 2). None of the individuals displayed abnormal bedside head impulse test results with naked eyes. The mean horizontal VOR gain was 0.99 ± 0.12, with no significant distinction between patients with and without DBS (0.99 ± 0.11 vs. 1.00 ± 0.11, *p* = 0.930). Comparable mean VOR gains were evident across all tested canals.

**Table 2:** Examination and vHIT findings in patients with PCS according to the presence of dorsal brainstem stroke.

	Total *n* = 80	With dorsal brainstem stroke *n* = 15	Without dorsal brainstem stroke *n* = 65	Value of *p*
**Examination**				
Spontaneous nystagmus	2 (2.5)	0 (0)	2 (3.1)	0.491
Skew deviation	6 (7.5)	4 (26.7)	2 (3.1)	0.002
EOM limitation	3 (3.8)	3 (20.0)	0 (0)	<0.001
Abnormal head impulse test	0 (0)	0 (0)	0 (0)	NA
Interval^*^, days	2 (1–3)	2 (1–3)	1 (1–3)	0.349
**Corrective saccades**				
Overt saccade	0 (0)	0 (0)	0 (0)	NA
Covert saccade	1 (1.3)	1 (6.7)	0 (0)	0.036
**vHIT gain**				
RH	1.00 ± 0.13	0.98 ± 0.10	1.00 ± 0.14	0.652
LH	1.00 ± 0.10	1.00 ± 0.12	1.00 ± 0.09	0.897
RP	0.98 ± 0.05	0.98 ± 0.02	0.98 ± 0.07	0.721
LA	0.98 ± 0.07	0.98 ± 0.06	0.98 ± 0.07	0.796
LP	0.96 ± 0.14	0.99 ± 0.03	0.96 ± 0.15	0.626
RA	0.97 ± 0.06	0.94 ± 0.12	0.98 ± 0.04	0.922
vHIT, abnormal gain^†^	1 (1.3)	1 (6.7)	0 (0)	0.036
vHIT GA (%)	4.3 ± 5.4	4.9 ± 4.1	4.2 ± 5.7	0.175

Results are presented as number and percent (% column) or mean ± SD or IQR. EOM, extraocular muscle; IQR, interquartile range; PCS, posterior circulation stroke; GA, gain asymmetry; IQR, interquartile range; LA, left anterior; LH, left horizontal; LP, left posterior; PCS, posterior circulation stroke; RA, right anterior; RH, right horizontal; RP, right posterior; SD, standard deviation; vHIT, video head impulse test. ^*^Between the onset of stroke symptoms or dizziness and vHIT. ^†^Abnormal vHIT gain was defined as a lateral gain lower than 0.8 for lateral canals and a posterior/anterior slope lower than 0.7 for anterior and posterior canals.

Abnormal VOR gain (0.70) in the right horizontal canal was identified in only one patient who had lesions in the vicinity of the MLF. Remarkably, this patient also presented with dysarthria, right-sided weakness, ataxia, and dizziness (Supplementary Table S1). Among the remaining 14 patients, VOR gains were not significantly abnormal even in patients who exhibited clinical symptoms of diplopia or neurological signs related to eye movements (e.g., internuclear ophthalmoplegia) or those with lesions near the central vestibular pathway (e.g., medial vestibular nucleus, vestibular nucleus, nucleus prepositus hypoglossi, and MLF). Regarding pathologic corrective saccades, overt saccades were absent in all cases. However, one patient who exhibited an abnormal VOR gain of 0.70 in the right horizontal canal demonstrated covert saccades.

Finally, we evaluated whether VOR gains differed among patients with DBS according to the rostrocaudal axis of lesion distribution. Five patients had midbrain lesions, whereas six and four patients had pontine and medullary lesions, respectively. Three patients had multiple lesion locations [two patients with pontine and cerebellar (PICA) lesions; one patient with medullary and cerebellar (PICA) lesions]. We found that VOR gains were similar for the midbrain, pons, and medulla groups (Supplementary Table S2), showing that VOR gains were not significantly different throughout the rostrocaudal axis of lesion distribution.

### Comparison between PCS with acute dizziness and VN

We extended our investigation to assess the diagnostic efficacy of vHIT in differentiating dizziness between PCS and VN. This analysis included only PCS patients with dizziness symptoms (*n* = 40). We then compared the vHIT results of these individuals with those of VN patients (*n* = 29) who had been previously examined at our institution. Of the VN patients, 27 (93.1%) underwent MRI confirming the absence of acute stroke lesions, while the remaining two showed significant unilateral vestibular hypofunction confirmed by caloric testing, without other neurological deficits suggestive of stroke. Patients with PCS exhibited vascular risk factors more often than those with VN (Table 3).

**Table 3:** Comparison baseline characteristics between PCS with acute dizziness and VN groups.

	PCS with acute dizziness *n* = 40	VN *n* = 29	Value of *p*
Age (years)	62 ± 13	57 ± 12	0.638
Male sex	29 (72.5)	18 (62.1)	0.359
Hypertension	27 (67.5)	10 (34.5)	0.007
Diabetes mellitus	16 (45.0)	2 (6.9)	0.001
Hyperlipidemia	20 (50.0)	2 (6.9)	<0.001
Atrial fibrillation	10 (25.0)	1 (3.4)	0.016
Smoking	16 (40.0)	0 (0)	<0.001
History of previous stroke	10 (25.0)	4 (13.8)	0.253
History of peripheral vertigo	0	2 (6.9)	0.092

Results are presented as number and percent (% column) or mean ± SD or IQR. PCS, posterior circulation stroke; VN, vestibular neuritis.

Clinically, the VN group displayed a higher incidence of spontaneous nystagmus and abnormal head impulse test results, while the PCS group exhibited skew deviation and restricted extraocular muscle movement (Table 4). In both groups, vHITs were conducted within a median of 2 days after symptom onset. Mean ipsilesional VOR gains were significantly lower across the assessed canals in the VN group compared to the PCS group. Furthermore, the percentage of patients displaying vHIT results with abnormal gain and VOR gain asymmetry was significantly higher in the VN group.

**Table 4:** Examination and vHIT Findings in PCS Patients with acute dizziness and those with VN.

	PCS with acute dizziness *n* = 40	VN *n* = 29	Value of *p*
**Examination**			
Spontaneous nystagmus	2 (5.0)	28 (96.6)	<0.001
Skew deviation	6 (15.0)	0 (0)	0.029
EOM limitation	3 (7.3)	0 (0)	0.132
Abnormal head impulse test	0 (0)	29 (100)	<0.001
Interval^*^, days	2 (1–4)	2 (1–4)	0.720
**Corrective saccades**			
Covert saccade	1 (2.5)	1 (3.4)	0.817
Overt saccade	0 (0)	28 (96.6)	<0.001
Saccades peak velocity	NA	237 ± 64	NA
**vHIT gain (ipsilesional)**			
Horizonal canal	1.00 ± 0.09	0.57 ± 0.32	<0.001
Posterior canal	0.97 ± 0.05	0.83 ± 0.19	<0.001
Anterior canal	0.97 ± 0.06	0.71 ± 0.26	<0.001
**vHIT gain (contralesional)**			
Horizonal canal	1.00 ± 0.09	1.03 ± 0.17	0.352
Posterior canal	0.98 ± 0.42	1.05 ± 0.15	0.115
Anterior canal	0.98 ± 0.07	1.06 ± 0.24	0.002
vHIT, abnormal gain^†^	1 (2.5)	25 (86.2)	<0.001
vHIT GA (%)	3.8 ± 4.0	32.8 ± 21.2	<0.001

Results are presented as number and percent (% column) or mean ± SD or IQR. EOM, extraocular muscle; GA, gain asymmetry; IQR, interquartile range; NA, not available; PCS, posterior circulation stroke; SD, standard deviation; vHIT, video head impulse test; VN, vestibular neuritis. ^*^Between the onset of stroke symptoms or dizziness and vHIT. ^†^Abnormal vHIT gain was defined as a lateral gain lower than 0.8 for lateral canals and a posterior/anterior slope lower than 0.7 for anterior and posterior canals.

**Figure 2:**
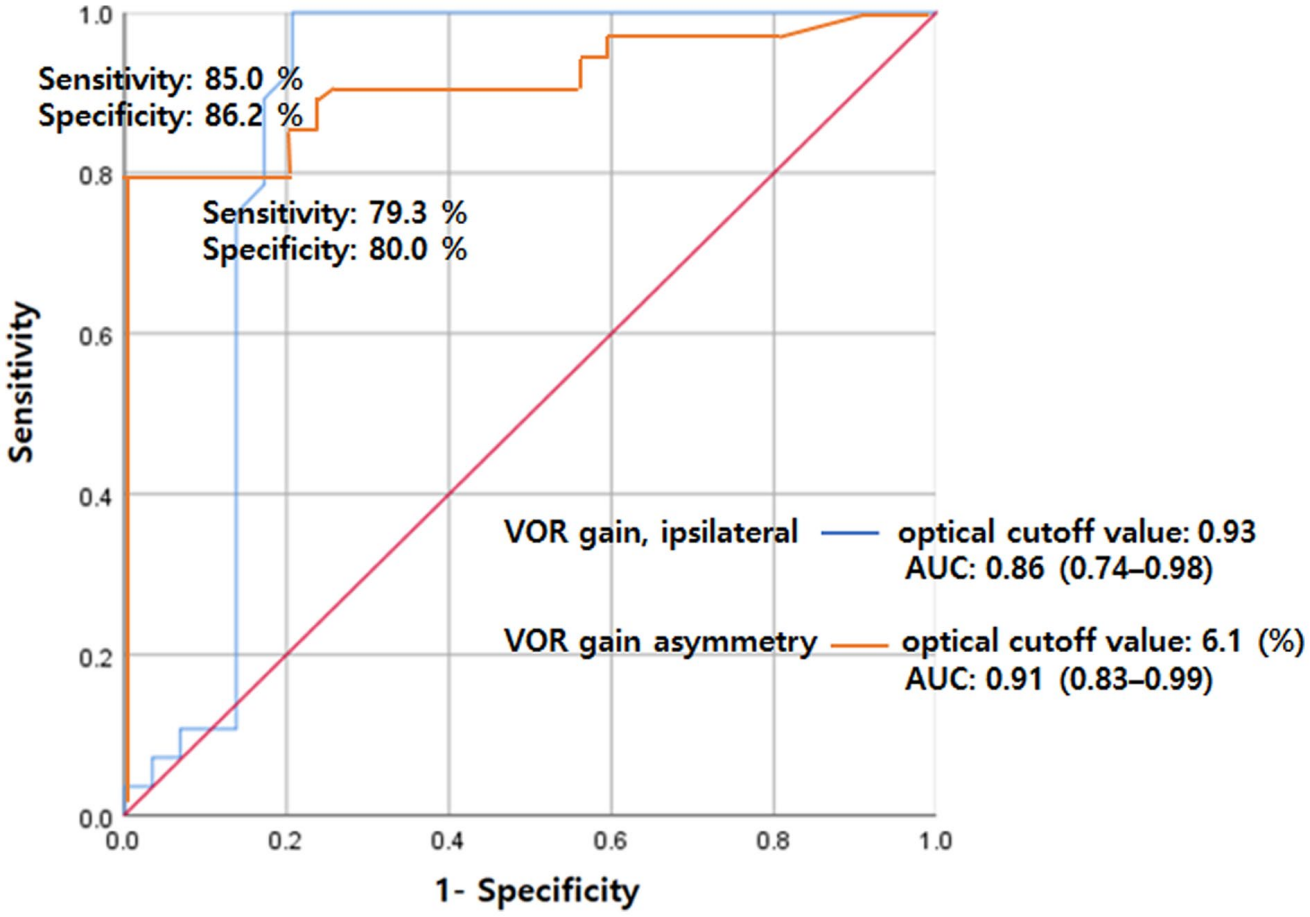
ROC analysis for VOR gain (ipsilateral) and gain asymmetry for separating VN from PCS. The sensitivity was 85.0% and specificity was 86.2% at cutoff values of 0.93 for VOR gain with an AUC of 0.86 (95% CI 0.74–0.98). Also, the sensitivity was 79.3% and specificity was 80.0% at a cutoff value of 6.1 for VOR asymmetry with an AUC of 0.91 (95% CI 0.83–0.99). AUC, area under the curve; PCS, posterior circulation stroke; ROC, receiver operating characteristics; VN, vestibular neuritis; VOR, vestibulo-ocular reflex.

Based on the ROC curves, VOR gain exhibited a sensitivity was 85.0% and specificity was 86.2% at cutoff values of 0.93, with an area under the curve (AUC) of 0.86 (95% CI 0.74–0.98, *p* < 0.001). Furthermore, VOR asymmetry displayed a sensitivity of 79.3% and specificity of 80.0% at a cutoff value of 6.1%, resulting in an AUC of 0.91 (95% CI 0.83–0.99, *p* < 0.001) (Figure 2).

## Discussion

In the present study, we prospectively investigated vHITs in patients with acute PCS within 24 h of symptom onset. VOR gains were not significantly affected in these patients, and they did not differ between the patients with and without DBS. Pathologic overt corrective saccades were absent in these patients, with only one individual exhibiting covert corrective saccades. Moreover, VOR gains as well as their asymmetry were effectively differentiated dizziness between PCS and VN. These findings underscore the potential value of vHIT as a diagnostic tool for acute dizziness.

Remarkably, when prospectively administered to acute PCS patients with mild symptoms, vHIT yielded infrequent significant abnormalities. Significant asymmetry and unidirectional corrective saccades are prominent in patients with VN (20, 21); thus, abnormal HIT findings have been suggested as an important sign to rule out central causes. However, the examination process *per se* may be technically demanding to non-experts, which decreases the clinical utility of this examination. Consequently, despite the high screening efficacy of the HINTs exam in excluding stroke, many emergency room physicians tend to place considerable reliance on costly imaging procedures like DWI (22). vHIT and its relevant application can serve as a valuable diagnostic approach, augmenting the objective measurement of VOR and supporting the HINTs exam (20). However, it has been untested prospectively whether vHIT findings are actually useful in patients with normal in terms of overt corrective saccades in patients with central causes of acute dizziness. In this regard, our results are notable and provide evidence for the concept of using vHIT as an ECG for the eyes in the emergency department. The clinical usefulness of vHIT will be further verified in the ongoing AVERT clinical trial (NCT02483429).

One patient with DBS presented pathologic covert corrective saccades and significantly decreased VOR gains. Lesions involving central VOR pathways may have affected the results in this patient. This finding is in line with several previous studies that showed unilaterally or bilaterally reduced VOR gains in patients with lesions involving the vestibular nucleus, nucleus prepositus hypoglossi, MLF, flocculus (8, 23), dorsolateral medulla, or cerebellar vermis (24). However, the degree of VOR decrease in patients with PCS was only modest in our case (0.70 for the right horizontal canal), as well as in the cited studies (0.88 ± 0.28) (24). Also, the degree of catch-up saccades in patients with stroke was much smaller than that of patients with VN (9, 25, 26). It should also be noted that the patient exhibited not only dizziness but also other stroke symptoms, such as dysarthria and right-sided weakness. Therefore, the diagnosis should be confirmed using other neurological examinations.

In terms of diagnostic performance, vHIT findings demonstrated remarkable success in distinguishing dizziness between VN and PCS. VOR gains and their asymmetry exhibited effectiveness in discerning the etiology of acute dizziness. The performance is comparable to or even surpasses results reported in previous studies (27, 28). These findings imply that VOR gains during vHIT and related parameters may offer valuable diagnostic insights into acute dizziness differentiation. These discoveries warrant validation in subsequent research and clinical application.

The rarity of pathologic vHIT findings even in patients with DBS with MLF lesions warrants further discussion. First, because the central VOR pathway ascends and spreads out bilaterally in the MLF from the medial vestibular nucleus (29), a focal lesion in this pathway may not materially affect the overall VOR process. Given the similar degrees of VOR gains for stroke lesions along the rostrocaudal axis of the brainstem, spreading of the VOR pathway may occur immediately after the vestibular nucleus and may change not substantially thereafter. As discussed above, patients with a considerable MLF lesion may present abnormal findings; however, the degree of vHIT abnormalities in these patients appeared to be modest. Second, vHIT may not be sensitive enough to detect subtle VOR changes in patients with stroke. Compared to search coils, vHIT is susceptible to recording artifacts and is less sensitive (30, 31). In the present study, if we had examined patients using magnetic search coils, abnormal VOR findings may have been detected. However, for a screening tool, being appropriately sensitive may be more helpful to distinguish between central and peripheral causes of acute dizziness, rather than being too sensitive. Moreover, patient discomfort associated with magnetic search coils presents another drawback in their role as a screening tool.

The strength of this study is its prospective design to systemically evaluate patients with PCS regardless of acute dizziness and the inclusion of supra/infratentorial ischemic lesions, such as those in the thalamus and midbrain. Most previous studies have focused on patients with acute dizziness and infratentorial lesions (32); however, dizziness is a vague symptom in patients with acute stroke and may even be present in those with supratentorial lesions (33, 34). Accordingly, several patients with supratentorial lesions [temporo-occipital area (*n* = 5, 16.1%), thalamus (*n* = 2, 6.5%), and midbrain (*n* = 2, 6.5%)] in the present study also had acute dizziness. For using vHIT as a screening tool in patients with acute vestibular syndrome, vHIT data must be collected from both stroke patients with infratentorial lesions and those with supratentorial lesions. Therefore, our results increase the applicability of vHIT for ruling out stroke in clinical practice.

Several limitations should be noted. First, we only included stroke patients with mild neurologic deficits (NIHSS score ≤ 7) for safety reasons, which may have led to the underestimation of the degree of VOR impairment in patients with stroke. However, patients who may mainly benefit from vHIT in the emergency department are those with mild neurologic deficits (e.g., only dizziness). Second, the interval between symptom onset to vHIT was a median of 2 days, and this delay may have affected the results reflecting acute situations in the emergency department (24). This was unavoidable as we tried to include all patients with PCS regardless of acute dizziness, as numerous patients with acute stroke require stabilization to safely undergo vHIT. Thus, we cannot negate the possibility of early abnormal vHIT findings that had normalized prior to vHIT. Moreover, half of the patients with acute strokes visit the emergency department after 24 h of symptom onset (35), and these patients are less likely to have severe strokes as they were brought to the emergency department. Considering that vHIT may be particularly useful in patients with mild stroke, our results still merit attention and should be considered clinically meaningful. Third, not all consecutive patients treated at our center were included for various reasons (Figure 1), which could have led to selection bias. Thus, our results should be interpreted with caution. Fourth, this study was performed at a single center, which limits the generalizability of its results. Specifically, the sample size for assessing lesion distribution in terms of VOR gains may not have been sufficient for conducting a statistically meaningful analysis. Finally, it should also be noted that the VN control group was not recruited in a prospective manner. Consequently, two out of 29 (6.9%) patients with VN did not undergo MRI to rule out stroke diagnosis, and the VN and PCS groups utilized different vHIT devices, despite adhering to the same protocol within the same institution.

Nevertheless, we found that vHITs in patients with acute PCS rarely show abnormal findings even in those with DBS. Importantly, we did not find pathologic overt corrective saccades in any studied patient with acute PCS; only one patient with DBS showed abnormal vHIT findings, such as covert corrective saccades and decreased VOR gains with a modest degree of abnormalities. Moreover, additional vHIT variables, such as VOR gains and their asymmetry, effectively distinguished dizziness between PCS and VN patients (Figure 2). These findings support the concept of using vHIT as a screening tool to differentiate between central and peripheral causes of acute dizziness in clinical settings.

## Data availability statement

The raw data supporting the conclusions of this article will be made available by the authors, without undue reservation.

## Author contributions

SH: Conceptualization, Data curation, Formal Analysis, Methodology, Writing – original draft. DL: Methodology, Writing – review & editing. GP: Data curation, Writing – review & editing. BK: Investigation, Methodology, Writing – review & editing. JC: Investigation, Methodology, Writing – review & editing. D-WK: Investigation, Methodology, Writing – review & editing. SK: Investigation, Methodology, Writing – review & editing. JK: Investigation, Methodology, Writing – review & editing. HP: Conceptualization, Data curation, Funding acquisition, Investigation, Methodology, Project administration, Resources, Supervision, Validation, Writing – original draft, Writing – review & editing. E-JL: Conceptualization, Data curation, Funding acquisition, Investigation, Methodology, Project administration, Resources, Supervision, Validation, Visualization, Writing – original draft, Writing – review & editing.

## References

[1] Newman-Toker DE Saber Tehrani AS Mantokoudis G Pula JH Guede CI Kerber KA. Quantitative video-oculography to help diagnose stroke in acute vertigo and dizziness: toward an ECG for the eyes. Stroke. (2013) 44:1158–61. doi: 10.1161/STROKEAHA.111.000033, PMID: 23463752 PMC8448203

[2] Lee H. Isolated vascular vertigo. J Stroke. (2014) 16:124–30. doi: 10.5853/jos.2014.16.3.124, PMID: 25328871 PMC4200599

[3] Kattah JC Talkad AV Wang DZ Hsieh YH Newman-Toker DE. HINTS to diagnose stroke in the acute vestibular syndrome: three-step bedside oculomotor examination more sensitive than early MRI diffusion-weighted imaging. Stroke. (2009) 40:3504–10. doi: 10.1161/STROKEAHA.109.551234, PMID: 19762709 PMC4593511

[4] Tarnutzer AA Berkowitz AL Robinson KA Hsieh Y-H Newman-Toker DE. Does my dizzy patient have a stroke? A systematic review of bedside diagnosis in acute vestibular syndrome. CMAJ Open. (2011) 183:E571–92. doi: 10.1503/cmaj.100174, PMID: 21576300 PMC3114934

[5] Machner B Erber K Choi JH Sprenger A Helmchen C Trillenberg P. A simple gain-based evaluation of the video head impulse test reliably detects Normal Vestibulo-ocular reflex indicative of stroke in patients with acute vestibular syndrome. Front Neurol. (2021) 12:741859. doi: 10.3389/fneur.2021.741859, PMID: 34777209 PMC8585749

[6] Black RA Halmagyi GM Thurtell MJ Todd MJ Curthoys IS. The active head-impulse test in unilateral peripheral Vestibulopathy. Arch Neurol. (2005) 62:290–3. doi: 10.1001/archneur.62.2.290, PMID: 15710858

[7] van Esch BF Nobel-Hoff GE van Benthem PP van der Zaag-Loonen HJ Bruintjes TD. Determining vestibular hypofunction: start with the video-head impulse test. Eur Arch Otorhinolaryngol. (2016) 273:3733–9. doi: 10.1007/s00405-016-4055-9, PMID: 27113255

[8] Choi JY Kim HJ Kim JS. Recent advances in head impulse test findings in central vestibular disorders. Neurology. (2018) 90:602–12. doi: 10.1212/WNL.0000000000005206 29490911

[9] Nam GS Shin HJ Kang JJ Lee NR Oh SY. Clinical implication of corrective saccades in the video head impulse test for the diagnosis of posterior inferior cerebellar artery infarction. Front Neurol. (2021) 12:605040. doi: 10.3389/fneur.2021.605040, PMID: 33679578 PMC7930369

[10] Chen L Todd M Halmagyi GM Aw S. Head impulse gain and saccade analysis in pontine-cerebellar stroke and vestibular neuritis. Neurology. (2014) 83:1513–22. doi: 10.1212/WNL.0000000000000906, PMID: 25253747 PMC4222852

[11] Liu YL Wu ZQ Qu JF Qiu DH Luo GP Yin HP. High neutrophil-to-lymphocyte ratio is a predictor of poor short-term outcome in patients with mild acute ischemic stroke receiving intravenous thrombolysis. Brain Behav. (2020) 10:e01857. doi: 10.1002/brb3.1857, PMID: 32981201 PMC7749577

[12] Lee EJ Nah HW Kwon JY Kang DW Kwon SU Kim JS. Ischemic stroke in patients with cancer: is it different from usual strokes? Int J Stroke. (2014) 9:406–12. doi: 10.1111/ijs.12124 23981525

[13] Yoo MH Yang CJ Kim SA Park MJ Ahn JH Chung JW. Efficacy of steroid therapy based on symptomatic and functional improvement in patients with vestibular neuritis: a prospective randomized controlled trial. Eur Arch Otorhinolaryngol. (2017) 274:2443–51. doi: 10.1007/s00405-017-4556-1, PMID: 28391531

[14] Karatas M. Central vertigo and dizziness: epidemiology, differential diagnosis, and common causes. Neurologist. (2008) 14:355–64. doi: 10.1097/NRL.0b013e31817533a3 19008741

[15] Kim BJ Kim JS. Ischemic stroke subtype classification: an asian viewpoint. J Stroke. (2014) 16:8–17. doi: 10.5853/jos.2014.16.1.8, PMID: 24741560 PMC3961817

[16] Lee SU Park SH Park JJ Kim HJ Han MK Bae HJ. Dorsal medullary infarction: distinct syndrome of isolated central Vestibulopathy. Stroke. (2015) 46:3081–7. doi: 10.1161/STROKEAHA.115.010972 26463694

[17] Yoo MH Kim SH Lee JY Yang CJ Lee HS Park HJ. Results of video head impulse and caloric tests in 36 patients with vestibular migraine and 23 patients with vestibular neuritis: a preliminary report. Clin Otolaryngol. (2015) 41:813–7. doi: 10.1111/coa.12556 26451523

[18] Calic Z Nham B Bradshaw AP Young AS Bhaskar S D’Souza M. Separating posterior-circulation stroke from vestibular neuritis with quantitative vestibular testing. Clin Neurophysiol. (2020) 131:2047–55. doi: 10.1016/j.clinph.2020.04.173 32600960

[19] Yang CJ Cha EH Park JW Kang BC Yoo MH Kang WS. Diagnostic value of gains and corrective saccades in video head impulse test in vestibular neuritis. Otolaryngol Head Neck Surg. (2018) 159:347–53. doi: 10.1177/0194599818768218, PMID: 29631490

[20] Kattah JC. Use of HINTS in the acute vestibular syndrome. An overview. Stroke Vasc Neurol. (2018) 3:190–6. doi: 10.1136/svn-2018-000160, PMID: 30637123 PMC6312070

[21] Newman-Toker DE Kattah JC Alvernia JE Wang DZ. Normal head impulse test differentiates acute cerebellar strokes from vestibular neuritis. Neurology. (2008) 70:2378–85. doi: 10.1212/01.wnl.0000314685.01433.0d, PMID: 18541870

[22] Quimby AE Kwok ESH Lelli D Johns P Tse D. Usage of the HINTS exam and neuroimaging in the assessment of peripheral vertigo in the emergency department. J Otolaryngol Head Neck Surg. (2018) 47:54. doi: 10.1186/s40463-018-0305-8, PMID: 30201056 PMC6131950

[23] Kim SH Kim HJ Kim JS. Isolated vestibular syndromes due to brainstem and cerebellar lesions. J Neurol. (2017) 264:63–9. doi: 10.1007/s00415-017-8455-6, PMID: 28314977

[24] Thomas JO Sharobeam A Venkat A Blair C Ozalp N Calic Z. Video head impulse testing to differentiate vestibular neuritis from posterior circulation stroke in the emergency department: a prospective observational study. BMJ Neurol Open. (2022) 4:e000284. doi: 10.1136/bmjno-2022-000284, PMID: 35571585 PMC9066478

[25] Park HK Kim JS Strupp M Zee DS. Isolated floccular infarction: impaired vestibular responses to horizontal head impulse. J Neurol. (2013) 260:1576–82. doi: 10.1007/s00415-013-6837-y, PMID: 23370610

[26] Machner B Erber K Choi JH Trillenberg P Sprenger A Helmchen C. Usability of the head impulse test in routine clinical practice in the emergency department to differentiate vestibular neuritis from stroke. Eur J Neurol. (2021) 28:1737–44. doi: 10.1111/ene.14707, PMID: 33382146

[27] Kim SH Lee SU Cho BH Cho KH Yu SW Kim BJ. Analyese of head-Impluse test in patients with posterior circulation stroke and vestibular neuritis. Neurology. (2023) 100:e2374–85. doi: 10.1212/WNL.0000000000207299 37076307 PMC10256120

[28] Guler A Karbek Akarca F Eraslan C Tarhan C Bilgen C Kirazli T. Clinical and video head impulse test in the diagnosis of posterior circulation stroke presenting as acute vestibular syndrome in the emergency department. J Vestib Res. (2017) 27:233–42. doi: 10.3233/VES-170620 29081427

[29] Ki-Bum Sung T-KL. Central mechanisms of the Vestibulo-ocular reflex (VOR). Korean Bal Soc. (2002) 1:55–66.

[30] Mantokoudis G Saber Tehrani AS Wozniak A Eibenberger K Kattah JC Guede CI. Impact of artifacts on VOR gain measures by video-oculography in the acute vestibular syndrome. J Vestib Res. (2016) 26:375–85. doi: 10.3233/VES-160587, PMID: 27814312 PMC6054448

[31] Macdougall HG McGarvie LA Halmagyi GM Curthoys IS Weber KP. The video head impulse test (vHIT) detects vertical semicircular canal dysfunction. PLoS One. (2013) 8:e61488. doi: 10.1371/journal.pone.0061488, PMID: 23630593 PMC3632590

[32] Oh SY Kim JS Lee JM Shin BS Hwang SB Kwak KC. Ocular vestibular evoked myogenic potentials induced by air-conducted sound in patients with acute brainstem lesions. Clin Neurophysiol. (2013) 124:770–8. doi: 10.1016/j.clinph.2012.09.026, PMID: 23121898

[33] Wijesinghe R Protti DA Camp AJ. Vestibular interactions in the thalamus. Front Neural Circuits. (2015) 9:79. doi: 10.3389/fncir.2015.00079 26696836 PMC4667082

[34] Lee E-J Kim H-J Kim J-S. Ocular, vestibular, and Otologic syndromes In: Kim JS, editor. Posterior circulation stroke: Advances in understanding and management. Singapore: Springer Singapore (2021). 101–19.

[35] Terecoasa EO Radu RA Negrila A Enache I Casaru B Tiu C. Pre-hospital delay in acute ischemic stroke care: current findings and future perspectives in a tertiary stroke center from Romania-a cross-sectional study. Medicina. (2022) 58:1003. doi: 10.3390/medicina58081003, PMID: 36013470 PMC9415394

