# Supplementary material for: Prospective analysis of video head impulse tests in patients with acute posterior circulation stroke
Source: Front Neurol. 2023 Sep 22;14:1256826. doi: 10.3389/fneur.2023.1256826 (PMC10557255; doi:10.3389/fneur.2023.1256826)
Supplement: Supplementary file 1 [file Table_1.DOCX]

**Table S1. VOR Gain, Demographics, and Topography in Patients With Dorsal Brainstem Infarction**

| **Case** | **Lesion** | **Clinical symptoms** | **Sex** | **Age** | **Stroke onset prior to vHIT (days)** | **RH** | **LH** | **RP** | **LA** | **LP** | **RA** |
| --- | --- | --- | --- | --- | --- | --- | --- | --- | --- | --- | --- |
| 1 | 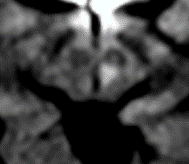midbrain (MLF) | vertical diplopia | M | 36 | 4 | 0.96 | 1.02 | 1.01 | 1.03 | 0.98 | 0.72 |
| 2 | 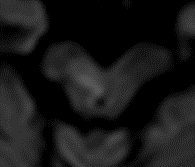midbrain (MLF) | left ataxia | M | 74 | 2 | 1.02 | 1.08 | NA | NA | NA | NA |
| 3 | 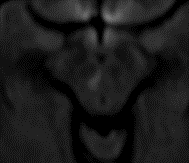midbrain (MLF) | vertical diplopia, dizziness | F | 51 | 1 | 1 | 0.96 | 1 | 1.05 | 0.92 | 0.77 |
| 4 | 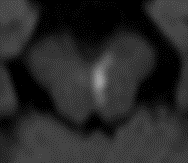midbrain (MLF) | vertical diplopia, dizziness | M | 66 | 0 | 1.01 | 1.06 | 0.99 | 0.96 | 1.01 | 0.98 |
| 5 | 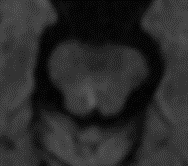pons (MLF) | right INO | M | 68 | 1 | 0.93 | 0.87 | 0.97 | 0.98 | 1.01 | 1.04 |
| 6 | 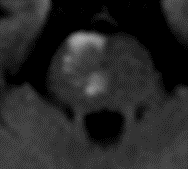pons | dizziness, left facial palsy, left-sided weakness | M | 64 | 1 | 1.02 | 0.95 | NA | NA | NA | NA |
| 7 | 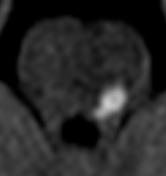pons | horizontal diplopia, ataxia | M | 61 | 3 | 1.04 | 1.02 | 0.99 | 1.03 | 0.98 | 0.98 |
| 8 | 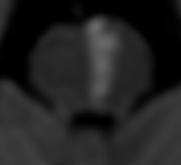pons (MLF) | left INO, dysarthria, left ataxia | M | 67 | 2 | 0.94 | 0.96 | 0.94 | 0.91 | 0.98 | 1.02 |
| 9 | 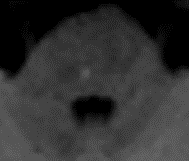pons | dysarthria | M | 79 | 2 | 1.12 | 0.89 | NA | NA | NA | NA |
| 10 | 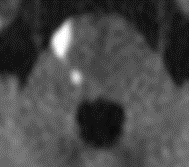pons | dysarthria, dizziness | F | 68 | 6 | 0.96 | 1.05 | NA | NA | NA | NA |
| 11 | 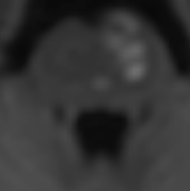pons (MLF) | dizziness, dysarthria, right-sided weakness | F | 76 | 1 | 0.70 | 0.91 | NA | NA | NA | NA |
| 12 | 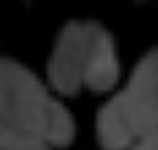medullar (VN) | dysarthria, dizziness, nausea, ataxia | F | 36 | 3 | 1.01 | 0.93 | 0.99 | 0.87 | 1.01 | 1 |
| 13 | 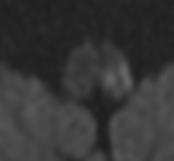medullar | sensory decline, ataxia | M | 60 | 4 | 0.82 | 0.98 | NA | NA | NA | NA |
| 14 | 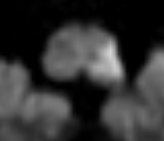 medullar (VN) | dizziness, ataxia | F | 75 | 3 | 1.09 | 1.37 | 0.98 | 0.99 | 0.99 | 1 |
| 15 | 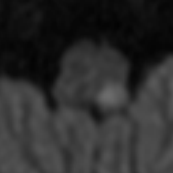medullar (VN) | sweating, dizziness, hoarseness, dysphagia, left facial sensory change, left ataxia | F | 48 | 3 | 1.01 | 0.98 | 0.99 | 0.99 | 1 | 0.99 |

F indicates female; INO, internuclear ophthalmoplegia; LA, left anterior; LH, left horizontal; LP, left posterior; M, male; MLF, medial longitudinal fasciculus; NA, not available; RA, right anterior; RH, right horizontal; RP, right posterior; vHIT, video head impulse test; VN, vestibular nucleus; VOR, vestibulo-ocular reflex.

**Table S2. VOR Gain in Patients with Dorsal Brainstem Stroke According to Caudal-to-Rostral Distribution**

|  | **Midbrain**  **(n = 5)** | **Pons**  **(n = 6)** | **Medulla**  **(n = 4)** | **Multiple lesions**  **(n = 3)*** |
| --- | --- | --- | --- | --- |
| RH | 0.98 ± 0.04 | 0.96 ± 0.14 | 0.98 ± 0.11 | 0.94 ± 0.22 |
| LH | 0.99 ± 0.08 | 0.96 ± 0.06 | 1.06 ± 0.20 | 0.91 ± 0.02 |
| RP | 0.99 ± 0.02 | 0.97 ± 0.04 | 0.98 ± 0.01 | 0.99 |
| LA | 1.01 ± 0.04 | 0.97 ± 0.08 | 0.95 ± 0.07 | 0.87 |
| LP | 0.98 ± 0.04 | 0.98 ± 0.00 | 1.00 ± 0.01 | 1.01 |
| RA | 0.88 ± 0.16 | 1.00 ± 0.03 | 0.99 ± 0.01 | 1.00 |

* Two patients with multiple lesions did not reliably undergo evaluation of vertical canals, resulting in a single value in VOR tests in vertical canals.

LA indicates left anterior; LH, left horizontal; LP, left posterior; RA, right anterior; RH, right horizontal; RP, right posterior; VOR, vestibulo-ocular reflex
